# Latent class analysis derived subgroups of low back pain patients – do they have prognostic capacity?

**DOI:** 10.1186/s12891-017-1708-9

**Published:** 2017-08-09

**Authors:** Anne Molgaard Nielsen, Lise Hestbaek, Werner Vach, Peter Kent, Alice Kongsted

**Affiliations:** 1 Department of Sports Science and Clinical Biomechanics, University of Southern Denmark, Campusvej 55, 5230 Odense M, Denmark; 2 Nordic Institute of Chiropractic and Clinical Biomechanics, University of Southern Denmark, 5230 Odense M, Denmark; 3 Institute for Medical Biometry and Statistics, Medical Center – University of Freiburg, Faculty of Medicine, University of Freiburg, 79104 Freiburg, Germany; 4 Department of Orthopaedics and Traumatology, University Hospital Basel, 4031 Basel, Switzerland; 5 School of Physiotherapy and Exercise Science, Curtin University, Perth, Australia

**Keywords:** Low back pain, Subgrouping, Classification, prognosis, Prospective studies, Latent class analysis

## Abstract

**Background:**

Heterogeneity in patients with low back pain is well recognised and different approaches to subgrouping have been proposed. One statistical technique that is increasingly being used is Latent Class Analysis as it performs subgrouping based on pattern recognition with high accuracy. Previously, we developed two novel suggestions for subgrouping patients with low back pain based on Latent Class Analysis of patient baseline characteristics (patient history and physical examination), which resulted in 7 subgroups when using a single-stage analysis, and 9 subgroups when using a two-stage approach. However, their prognostic capacity was unexplored. This study (i) determined whether the subgrouping approaches were associated with the future outcomes of pain intensity, pain frequency and disability, (ii) assessed whether one of these two approaches was more strongly or more consistently associated with these outcomes, and (iii) assessed the performance of the novel subgroupings as compared to the following variables: two existing subgrouping tools (STarT Back Tool and Quebec Task Force classification), four baseline characteristics and a group of previously identified domain-specific patient categorisations (collectively, the ‘comparator variables’).

**Methods:**

This was a longitudinal cohort study of 928 patients consulting for low back pain in primary care. The associations between each subgroup approach and outcomes at 2 weeks, 3 and 12 months, and with weekly SMS responses were tested in linear regression models, and their prognostic capacity (variance explained) was compared to that of the comparator variables listed above.

**Results:**

The two previously identified subgroupings were similarly associated with all outcomes. The prognostic capacity of both subgroupings was better than that of the comparator variables, except for participants’ recovery beliefs and the domain-specific categorisations, but was still limited. The explained variance ranged from 4.3%–6.9% for pain intensity and from 6.8%–20.3% for disability, and highest at the 2 weeks follow-up.

**Conclusions:**

Latent Class-derived subgroups provided additional prognostic information when compared to a range of variables, but the improvements were not substantial enough to warrant further development into a new prognostic tool. Further research could investigate if these novel subgrouping approaches may help to improve existing tools that subgroup low back pain patients.

**Electronic supplementary material:**

The online version of this article (doi:10.1186/s12891-017-1708-9) contains supplementary material, which is available to authorized users.

## Background

Low back pain (LBP) is highly prevalent and has several negative consequences for society and individual patients [1, 2]. In order to increase understanding of this health condition, to enable better estimates of prognosis, and to better match treatment to relevant characteristics of the patients, several studies of various approaches to subgrouping (or classification) have been conducted [3–8]. However, much of the heterogeneity in LBP patients remains poorly understood [9].

Many of the existing subgrouping approaches for use in primary care have been developed using a quite narrow spectrum of patient characteristics [10–16], for example, the Quebec Task Force classification, which represents different degrees of back pain with or without the presence of leg pain and signs of nerve root involvement [7]. This classification is a diagnostic tool but has shown some predictive ability [17, 18]. Other subgrouping approaches have included a broader spectrum of characteristics in their subgroups, but aimed at identifying a specific number of subgroups related to a particular treatment approach [3, 19, 20] or treatment need [5, 21]. For example, the STarT Back Tool, which is a stratification tool for treatment pathways, was developed using a method that drew upon the domains of pain, participation, activity, psychology, and the contextual factors of sex and age [5]. Its final form is a 9-item questionnaire stratifying patients into three risk groups of poor outcome from the current LBP episode, based on factors from the domains of pain, activity and psychology. The tool aims at improving health outcomes and healthcare cost savings by targeting potentially modifiable factors within each risk group [22].

These and other subgrouping approaches have increased our understanding of LBP but their prognostic capacity is still limited [23–26], and treatments based on subgroupings have had small to moderate effects [10, 11, 13–15, 22, 27, 28]. Therefore, the investigation of new approaches to subgrouping seems to have a merit.

One aspect to consider is that most previous subgrouping approaches have used traditional regression modelling techniques in which the multivariable relationships between the included variables are assumed to be the same for all people in the sample. However, one of the theories underlying subgrouping is that these multivariable relationships might vary, with one subgroup having one set of relationships between these variables and another subgroup having a different set of multivariable relationships. For example, hypothetically there may be a strong relationship in one subgroup between fear of movement and activity limitation, whereas this relationship may be weak or absent in another subgroup with different patient characteristics. It is likely that the essential information about prognosis and response to treatment does not lie in single variables but in certain scoring patterns across several variables, and also possible that these scoring patterns may differ across subgroups of patients. The identification of such subgroups may be a key to a better understanding and management of LBP patients. Classifying the population of LBP patients into such subgroups may also address the issue that the relationship between the outcome and some baseline variables may differ dependent on other baseline variables and that subgroup membership may be one of those variables [29].

A recent study [30] attempted to address these issues using Latent Class Analysis (LCA), as LCA allows exploration of how multidimensional baseline variables might form distinct patient subgroups when these are not assumed to be related to each other in the same way in all patients [31]. In that study, 112 baseline characteristics covering aspects of activity, contextual factors (such as sex, age, educational level and comorbidity), pain, participation, physical impairment and psychology were used in a LCA to identify subgroups of patients. One subgrouping was formed using a traditional single-stage approach to LCA, where all baseline variables were included simultaneously in the subgrouping analysis. The other subgrouping was formed from a two-stage approach, where the baseline variables were descriptively classified into six health domains, then participants were initially subgrouped by LCA within each of these domains to identify ‘domain-specific patient categorisations’ [32] and, subsequently these subgroup memberships were used in a second LCA to identify subgroups across domains [33, 34]. The use of health domains was an attempt to potentially increase the interpretability of the identified subgroups [33]. In both LCA approaches, clinical interpretable subgroups which differed on several baseline features were identified, but neither approach (single-stage or two-stage) was judged superior to the other based on statistical measures and face validity.

So, this study explored whether these two novel subgrouping approaches have any clinical significant difference in their capacity for improving prognostic estimates, as a mechanism to determine whether this aspect of external validity differed between the two subgrouping approaches. Therefore, the aim of this paper was to (i) determine whether membership in these previously identified subgroups was associated with outcomes of pain intensity, pain frequency and disability over the 12 months after the baseline consultation, (ii) assess whether one of these two subgroup models was more strongly and/or consistently associated with these outcomes, and (iii) assess the prognostic performance of these novel models compared to two existing subgrouping tools, four baseline characteristics that are known to have some prognostic capacity and to previously identified domain-specific patient categorisations.

## Methods

### Design and setting

This study used data from a prospective observational cohort study which included adult patients with LBP who had consulted one of 17 chiropractic clinics in the research network of the Nordic Institute for Chiropractic and Clinical Biomechanics in Denmark between September 2010 to January 2012. The patients were followed for 1 year during which interventions were not influenced by participation in the study. Further information about the cohort study has been reported previously [35–37]. Consenting participants with data on both patient self-reported and clinician-reported baseline questionnaires were included in the current study and their follow-up data, based on self-reported questionnaires (2 weeks, 3 and 12 months) and weekly Short Message Service (SMS) cell phone questions over 12 months, were used as measures of patient outcomes.

### Study population

Included patients had a main complaint of LBP with or without leg pain, were between 18 and 65 years old, could adequately read and write Danish, had a mobile phone and were able to send a text message. The patients were included regardless of the duration of their LBP episode as our pragmatic interest was in subgroups that could be found within the broad spectrum of cases that present in primary care and because episode duration was one of the inputs to the LCA it could influence subgroup formation. Prospective participants were excluded if they were pregnant, had serious pathology including pathology of the back that necessitated referral for acute surgical assessment or had attended more than one consultation for LBP in the preceding 3 months. The latter was to establish a cohort of patients who were included at the time point when first seeking care for an episode of LBP.

In total, 947 patients fulfilled the inclusion criteria and provided written consent. For 928 of those, both the patient- and clinician-reported questionnaires were completed at baseline, and therefore they were included in the current study. The available sample size was not based on the requirements of the current study, but was nonetheless adequate for applying regression models to test the statistical association between an outcome and many independent variables simultaneously, using the typical rule-of-thumb that a minimum of ten participants per independent variable is required or other related rules [38].

Participation in this study did not affect treatment and accordingly under Danish law, no ethical approval was needed [39]. Approval was obtained from the Danish Data Protection Agency (ref. no. 2012–41-0762).

### Comparator prognostic variables

#### Subgroupings derived by latent class analysis

The main focus in this study was the subgroup membership in the two subgroupings previously identified by way of two separately conducted LCAs [30], one using a traditional single stage approach and another using a two-stage approach [33, 34]. No imputations were performed during the subgrouping analyses, as LCA uses a likelihood approach which accommodates the inclusion of patients with missing values. In the previous study, the single-stage LCA approach included 112 baseline characteristics that were modelled simultaneously to identify ‘single-stage subgroups’. In the two-stage LCA approach, the 112 baseline characteristics were classified into six mutually exclusive health domains that were used initially to identify ‘domain-specific patient categorisations’ (first stage LCA) and these were then used as the input to a second stage LCA that finally identified ‘two-stage subgroups’ across domains. This resulted in seven single-stage subgroups and nine two-stage subgroups. The selection of the preferred subgroup solutions for each LCA approach had been informed by both statistical performance measures and a qualitative evaluation of clinical interpretability (face validity) [30]. The qualitative evaluation emphasised differences between the subgroups that were not only on a continuum of severity, but distinct differences in scoring patterns across the baseline characteristic. For example, a qualitative difference of two subgroups displaying opposite scoring patterns on the same variables. Using this approach, potentially novel relationships between the baseline characteristics were more likely to be identified. A brief description of each subgroup is presented in Table 1 and a thorough description was previously published [30]. Within the analyses in this paper, each subgroup solution was used as a categorical variable. The chosen order of the categories was based on severity level as determined by the baseline description of each subgroup. All authors contributed to this process and the final order was reached by consensus.

**Table 1:** Prevalence of Latent Class Analysis derived subgroups

	Prevalence, N
Single-stage subgroups	*N* = 928
SS1: Mildly affected: mild intermittent LBP (mild)	154 (17%)
SS2: Recent onset severe LBP, activity limitations (recent disability)	192 (21%)
SS3: Pain- and work-related concerns, high physical workload (work-related)	130 (14%)
SS4: Nerve root involvement (nerve root)	75 (8%)
SS5: Severely affected: very recent onset severe LBP, social participation and activity limitations (very recent)	136 (15%)
SS6: Persistent LBP, psychological issues, activity limitations and comorbidity (persistent)	132 (14%)
SS7: Severely affected: recent onset LBP with several consequences (severe)	109 (12%)
Two-stage subgroups	*N* = 928
TS1: Mildly affected: mild intermittent LBP, moderate activity limitations, no participation limitations (low degree of physical workload) (mild)	161 (17%)
TS2: Mildly affected: mild intermittent LBP with work-issues, no activity limitations, males (mild, work issues)	74 (8%)
TS3: Mildly affected: mild intermittent LBP, sleeps well, moderate activity limitations and sacroiliac joint pain, more females (mild, sleep well)	69 (7%)
TS4: Mildly affected: persistent LBP with sacroiliac joint pain (mild persistent)	45 (5%)
TS5: Recent onset severe LBP, activity limitations, sleep issues (sleep issues)	113 (12%)
TS6: Work-related severe LBP (work-related)	127 (14%)
TS7: Nerve root involvement (nerve root)	49 (5%)
TS8: Severely affected: very recent onset severe LBP, social participation and activity limitations (very recent)	219 (24%)
TS9: Severely affected: LBP with several consequences (severe)	71 (8%)

*LBP* low back pain

In this paper, the six independent domain-specific patient categorisations (from the first stage of the two-stage approach) were also used (See Additional file 1: Table A1). The derivation of four of these categorisations has been reported in another study that compared different LCA strategies for using questionnaire data [32].

#### Baseline characteristics included in the comparison of prognostic capacity

Two other subgrouping systems were used as examples of the prognostic capacity of existing subgroupings. One was the STarT Back Tool (SBT), which has three subgroups that are based on patients’ risk of persistent LBP disability (low, medium, high risk subgroups) calculated from a patient self-reported questionnaire [5, 22, 40]. The other was the Quebec Task Force classification (local LBP only, LBP + leg pain above the knee, LBP + leg pain below the knee, and LBP + leg pain and neurological signs) representing a simple measure based on the clinician’s clinical assessment [7, 17].

Four simple and commonly used baseline variables known to have some prognostic capacity were also used for the comparisons of prognostic capacity. These were: typical LBP and typical leg pain intensity during the preceding week measured by 0 to 10 Numeric Rating Scales [41, 42], the Danish 23 item version of the Roland-Morris Disability Questionnaire (RMDQ-23) used as a measure of pain-related disability that was proportionally recalculated to a 0–100 score where 0 corresponding to ‘no disability’ and 100 ‘highest disability’ [43, 44], and participants’ recovery belief measured by a Numeric Rating Scale with 0 equalling ‘not at all likely to recover’ and 10 ‘very likely to recover’ [37, 45].

### Outcome measures

Two outcomes at 2 weeks, 3 and 12 months were collected by post using patient self-completed questionnaires: LBP intensity (typical LBP the last week, 0–10 Numeric Rating Scale) and pain-related disability (the proportionally recalculated score from the RMDQ-23). Three other outcome measures were collected via weekly responses over 12 months using automated SMS, with participants replying with a number to each of the following questions 1) days with LBP the last week (0–7), 2) typical pain intensity the last week (0–10) and, 3) days with activity limitation the last week (0–7) [46, 47]. The second and third questions were sent if the participants replied with other than a zero to the first question.

### Data analysis

In the descriptive analysis, nominal scale variables were presented as proportions and continuous scale variables as medians and their interquartile range. Baseline comparisons between the single-stage and two-stage LCA subgroups, between responders and non-responders at the three follow-up time points, as well as comparisons between compliant SMS responders (less than 10 missing weeks of SMS replies [47]) and non-compliant responders, were tested using χ^2^ test for nominal variables, and Kruskal-Wallis equality-of-populations rank test and Wilcoxon rank-sum test (Mann–Whitney U test) for ordinal and continuous variables in relation to subgroups and responders, respectively.

#### Association with outcome

The distribution of scores from each subgroup at every follow-up time point were visualised and graphically compared using stacked bar charts (pain intensity) and box plots (disability). Linear regression modelling with robust variance estimations was used to analyse if the subgroups were significantly associated with the outcome measures at 2 weeks, 3 months and 12 months follow-ups. In addition, illustrations of course trajectories that showed the mean score per week over 12 months for each subgroup were visually compared between the subgroups for each of the three SMS outcome measures.

The significance of the differences between subgroups were assessed by a longitudinal linear regression model with the time in weeks as well as the subgroups as categorical covariates, taking the within-patient clustering (due to 52 weekly SMS responses being modelled for each patient) into account by using robust variance estimation to compute *p*-values. No pairwise comparison were performed. The visually observed differences between the mean trajectories were summarized across the three outcomes verbally.

#### Prognostic capacity

Prognostic capacity was measured by the amount of variance explained in outcomes and quantified by the adjusted R^2^ obtained in linear regression models, which was used for comparisons between the LCA-derived subgroupings and the previously described baseline characteristics. In addition, we considered the adjusted R^2^ from a regression model that included the six domain-specific patient categorisations as categorical covariates: this way we could assess whether we lost prognostic information in the second stage of the two-stage approach.

To explore if the LCA-derived subgroupings added prognostic capacity to that of the baseline values of the outcome measures of pain intensity and disability, or to that of participants’ recovery beliefs, we used linear regression models to calculate the difference in adjusted R^2^ obtained from models including only these baseline variables, and models in which the subgrouping variable was added. Although the inclusion of the baseline values is conceptually the same as analysing change scores, we chose to include the baseline values as these are available for the clinician when considering the prognosis of the individual patients, and change scores are unknown. Lastly, we calculated the additional prognostic capacity after taking into account both the baseline value of the respective outcome variables and the participants’ recovery beliefs. Again, for each of the investigated models, the added prognostic capacity of the LCA-derived subgroupings was compared to that of the two other subgrouping tools, the remaining baseline variables and the domain-specific patient categorisations.

All subgroup types (single-stage subgroups, two-stage subgroups, SBT, Quebec classification and domain-specific patient categorisations) were entered as categorical variables in the regression analysis. The four simple baseline variables were entered as continuous variables. In all tests of associations, a *P* value <.05 was considered significant.

#### Statistical software

All analyses were performed using STATA/IC 14.1 (StataCorp LP, College Station, TX, USA).

## Results

Females were 45% of the participants, the mean age was 43 and the most common duration of LBP before consulting the chiropractor was 2 weeks or less (62%) (Table 2). On all of the tested baseline characteristics, significant differences were found between the subgroups derived from the single-stage, as well as between those derived using the two-stage approach (See Additional file 1: Table A2a, for the baseline characteristics of the single-stage subgroups and Table A2b for the baseline characteristics of the two-stage subgroups). The differences were typically small to moderate in size, except of those which you would expect based on the definition of the groups. For example, the median leg pain intensity ranged from 0 to 2 across most subgroups but was higher in subgroups defined by nerve root involvement (subgroups SS4 and TS7, median = 6) and in the most severe subgroups with several consequences (SS7 and TS9, median = 4 and 3, respectively).

**Table 2:** Baseline characteristics of the low back pain cohort

	Low back pain patients *N* = 928
Males, N	510 (55%)
Age in years, median (interquartile range)	43 (34–53)
Highest achieved education, N	
No qualification	81 (9%)
Vocational training	236 (25%)
Higher education <3 years	142 (15%)
Higher education 3–4 years	311 (34%)
Higher education >4 years	136 (15%)
Missing	22 (2%)
Episode duration, N	
0–2 weeks	571 (62%)
2–4 weeks	123 (13%)
1–3 months	95 (10%)
> 3 months	121 (13%)
Missing	18 (2%)
Back pain intensity (0–10 Numeric Rating Scale), median (interquartile range)	7 (5–8)
Missing, N	25 (3%)
Leg pain intensity (0–10 Numeric Rating Scale), median (interquartile range)	2 (0–4)
Missing, N	43 (5%)
STarT Back Tool score, N	
Low risk	497 (54%)
Medium risk	351 (38%)
High risk	72 (8%)
Missing	8 (1%)
Quebec classification, N	
Local low back pain only	609 (66%)
Low back pain + leg pain above the knee	218 (23%)
Low back pain + leg pain below the knee	69 (7%)
Low back pain + leg pain and neurological signs	20 (2%)
Missing, N	12 (1%)
Roland-Morris Disability Questionnaire, proportionally recalculated score (0–100 = highest disability), median (interquartile range)	52 (35–70)
Missing, N	14 (2%)
Recovery belief (0–10 = very likely), median (interquartile range)	9 (7–10)
Missing, N	13 (1%)

### Response rates

The response rates to the follow-up questionnaires varied across outcomes and follow-up time points with the lowest rates on any outcome being 67% at 2 weeks, 79% at 3 months and 73% at 12 months. Baseline differences between responders and non-responders were similar across outcomes and time points with non-responders being younger, more often males and having slightly lower recovery beliefs (See Additional file 1: Table A3 which presents the comparisons between responders and non-responders). For example, for the outcome of disability at 12 months follow-up, non-responders were younger [39 years versus 45 years, *P* < .01]; more often male [62% versus 53%, *P* = .01], and had slightly lower recovery beliefs [median 9 {IQR 5–10} versus median 9 {IQR 7–10}, *P* = .01]. Within each of the single-stage and two-stage approaches, the response rates differed between subgroups at 12 months for the questionnaire outcomes of LBP intensity and disability, with non-responders being slightly overrepresented in the following single-stage subgroups: SS3 (work-related), SS6 (persistent) and SS7 (severe), and two-stage subgroups: TS6 (work-related), TS7 (nerve root), TS8 (very recent) and TS9 (severe).

There was a high response rate across the whole year to the SMS questions with 80% of individuals having less than 10 weeks with missing SMSs (compliant responders), and 76% of the participants responded to the SMS questions in all of the last 3 weeks (week 50 to week 52). The baseline differences between the compliant and non-compliant SMS responders were similar across the three SMS questions (See Additional file 1: Table A4, which presents the comparisons between compliant responders and non-compliant responders). For the outcome of LBP intensity, the non-compliant responders were underrepresented in the SBT low risk group (45% versus 56%, *P* = .01), had a slightly higher baseline leg pain intensity (2 versus 1, *P* = .07) and slightly lower recovery beliefs (median 9 [IQR 5–10] versus median 9 [IQR 7–10], *P* = .08). Only the single-stage subgroups differed statistically in their response rates with non-compliant responders being slightly underrepresented in the SS2 (recent disability) and SS4 (nerve root) subgroups.

### Associations between the single-stage subgroups and outcomes

#### Pain intensity

**Fig. 1:**
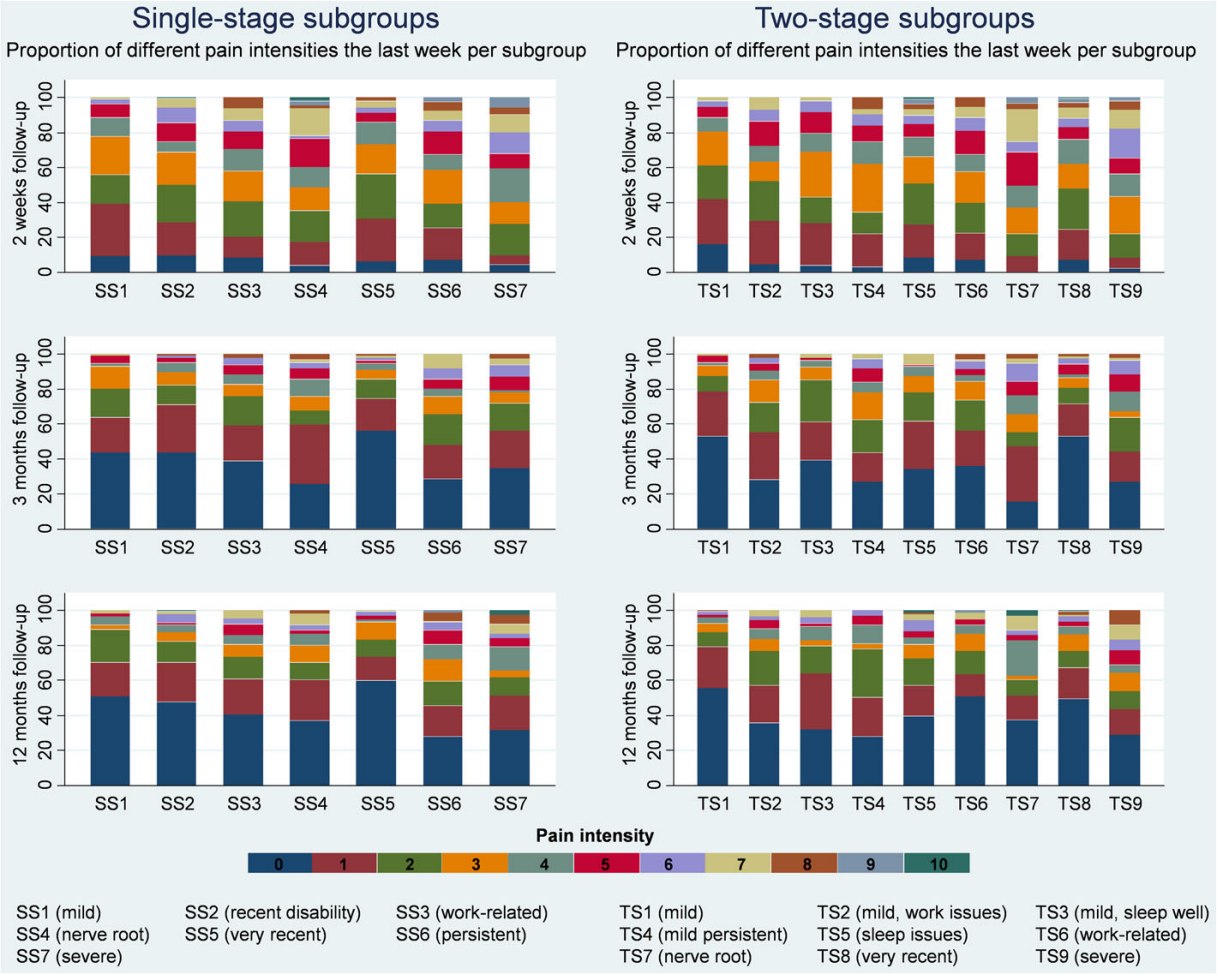
Pain intensity for the two subgrouping approaches. SS = single-stage subgroups; TS = two-stage subgroups. Proportion of patients reporting each level of typical LBP intensity within the last week (0–10) at 2-weeks, 3-months and 12-months follow-up

There was a statistically significant association between single-stage subgroups and pain at all three outcome time points (Table 3). Generally, the patients in subgroups SS1 (mild), SS2 (recent disability) and SS5 (very recent) had the best outcome in terms of reporting low absolute pain scores over time, the patients in subgroups SS6 (persistent) and SS7 (severe) reported the worst outcome, and SS3 (work-related) and SS4 (nerve root) patients had intermediate pain scores (Fig. 1).

**Table 3:** Prognostic capacity for absolute outcomes

			Follow-up time points		
Outcome measure	Description of prognostic variable	Prognostic variable	2 weeks	3 months	12 months
			Adj. R^2^	Adj. R^2^	Adj. R^2^
Pain intensity (Numeric Rating Scale, 0–10)	LCA-derived subgroupings	Single-stage subgroups	0.069**	0.043**	0.063**
		Two-stage subgroups	0.065**	0.052**	0.047**
	Existing subgrouping tools	STarT Back Tool	0.037**	0.012*	0.017*
		Quebec Task Force classification	0.018*	0.016*	0.008
	Baseline characteristics	Baseline back pain intensity (0–10 Numeric Rating Scale)	0.030**	0.007*	0.007*
		Baseline leg pain intensity (0–10 Numeric Rating Scale)	0.062**	0.016**	0.043**
		Baseline disability (RMDQ-23) (0–100 = highest disability)	0.024**	−0.001	0.004*
		Recovery belief (0–10 = very likely)	0.065**	0.109**	0.065**
	Results of first stage of two-stage LCA	All domain-specific patient categorisations	0.099**	0.126**	0.136**
Disability (23-item Roland Morris Disability Questionnaire, proportional score)	LCA-derived subgroupings	Single-stage subgroups	0.203**	0.113**	0.084**
		Two-stage subgroups	0.185**	0.113**	0.068**
	Existing subgrouping tools	STarT Back Tool	0.119**	0.079**	0.042**
		Quebec Task Force classification	0.042**	0.052**	0.042**
	Baseline characteristics	Baseline back pain intensity (0–10 Numeric Rating Scale)	0.056**	0.011*	0.005*
		Baseline leg pain intensity (0–10 Numeric Rating Scale)	0.062**	0.053**	0.057**
		Baseline disability (RMDQ-23) (0–100 = highest disability)	0.193**	0.060**	0.045**
		Recovery belief (0–10 = very likely)	0.030**	0.107**	0.110**
	Results of first stage of two-stage LCA	All domain-specific patient categorisations	0.259**	0.199**	0.186**

*Adj*. adjusted, *LCA* Latent Class Analysis

*P < .05

**P < .001

#### Trajectories

**Fig. 2:**
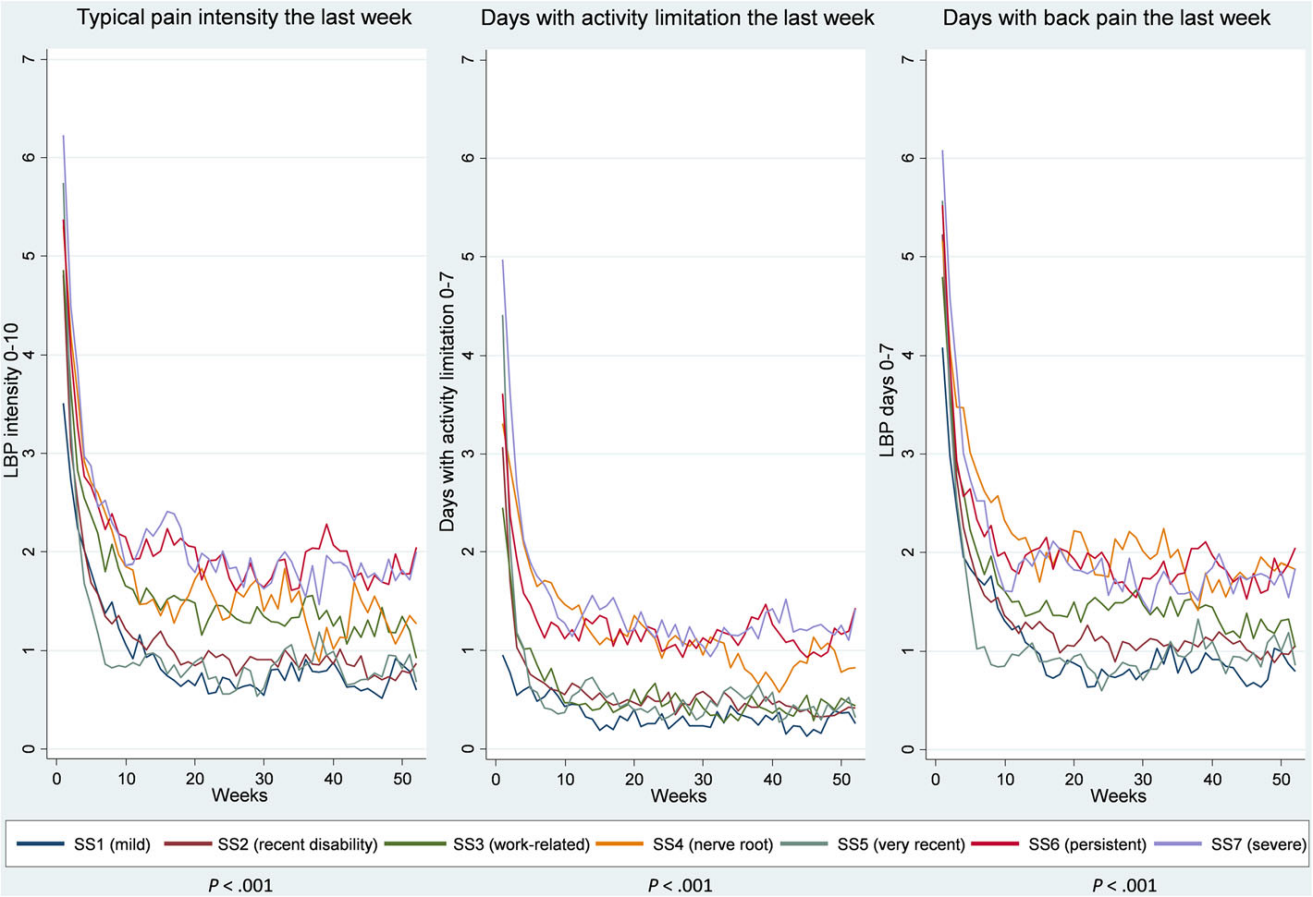
Trajectories based on weekly SMS questions over 12 months for the single-stage subgroups. SMS: Short Message Service; LBP = low back pain; SS = single-stage subgroups; *p*-values indicate a statistical significant difference between the subgroups for that outcome

For all three types of outcome trajectories (pain intensity, pain frequency, and disability frequency) we found statistically significant differences between the single-stage subgroups and the outcome (*p* < 0.001). The patients in subgroups SS1 (mild), SS2 (recent disability) and SS5 (very recent) had the mildest trajectories across these SMS outcomes and the SS3 (work-related) patients differed from them by having had a higher intensity and frequency of LBP. The patients in subgroups SS4 (nerve root), SS6 (persistent) and SS7 (severe) had the most severe trajectories although SS4 (nerve root) patients reported slightly less intense LBP than the other two subgroups (Fig. 2).

#### Disability

**Fig. 3:**
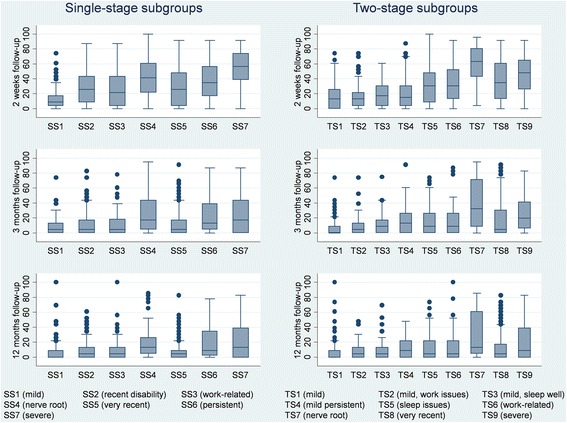
Roland-Morris proportionally recalculated disability score for the two subgrouping approaches. SS = single-stage subgroups; TS = two-stage subgroups

Statistically significant associations were found between the single-stage subgroups and disability at all three follow-up time points (Table 3). Generally, SS1 (mild), SS2 (recent disability), SS3 (work-related) and SS5 (very recent) patients had the lowest disability scores, followed by SS4 (nerve root) and SS6 (persistent) patients. The SS7 (severe) patients reported the highest disability score, however they experienced a slower but eventually better recovery compared to SS4 (nerve root) and SS6 (persistent) patients (Fig. 3).

Across all outcomes the general order of the subgroups on outcome severity was SS1 (mild), SS2 (recent disability), SS5 (very recent), SS3 (work-related), SS4 (nerve root), SS6 (persistent) and SS7 (severe).

### Associations between the two-stage subgroups and outcomes

#### Pain intensity

There was a statistically significant association between the two-stage subgroups and pain intensity at all three outcome time points (Table 3). Overall, TS1 (mild) patients had the lowest pain intensity score and TS7 (nerve root) and TS9 (severe) patients had the highest pain intensity score. The patients in the remaining subgroups had intermediate pain scores and differed only slightly, such as TS4 (persistent) patients having the slowest recovery rate and TS8 (very recent) patients having the fastest recovery, compared to the others (Fig. 1).

#### Trajectories

**Fig. 4:**
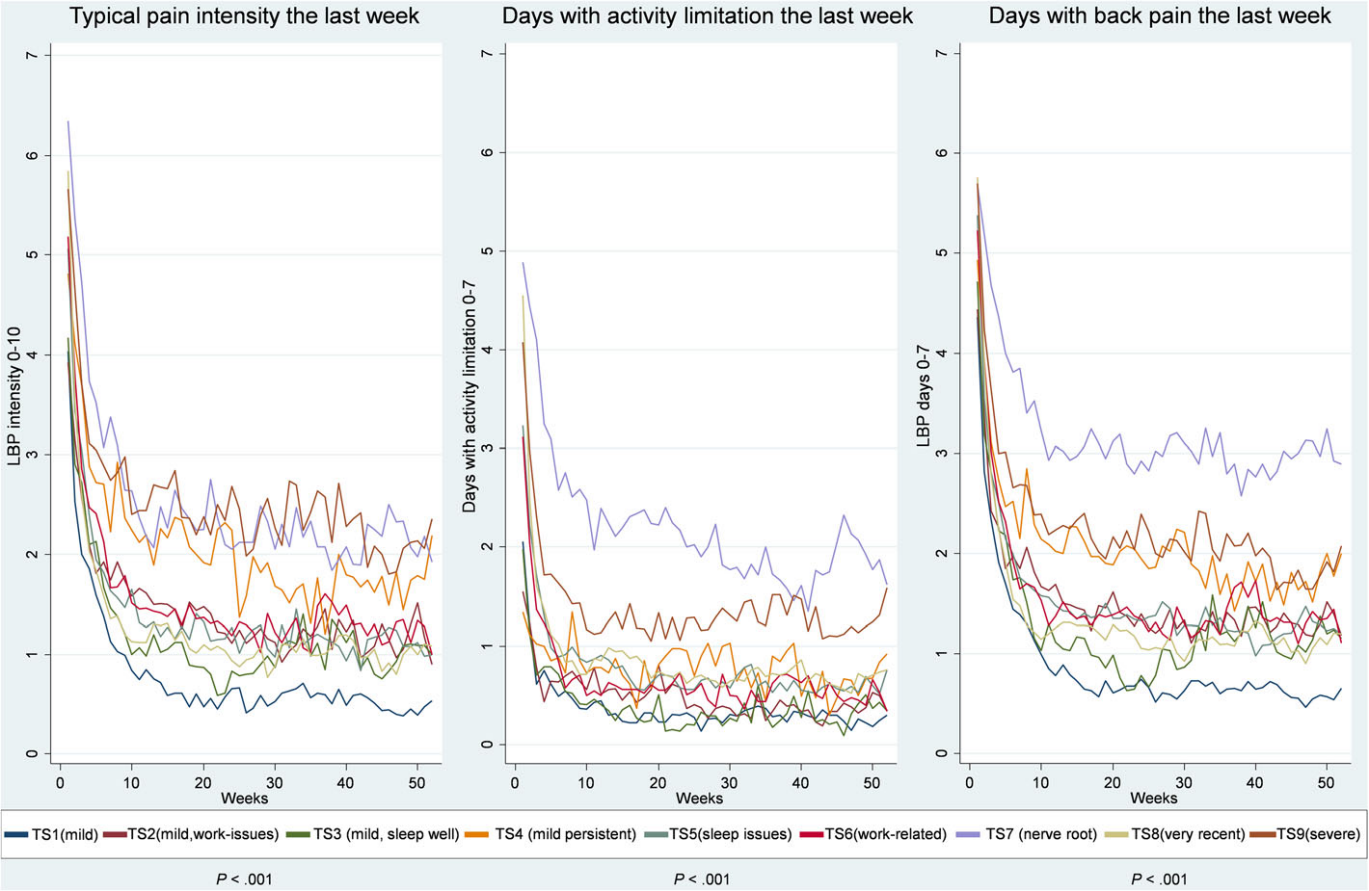
Trajectories based on weekly SMS questions over 12 months for the two-stage subgroups. SMS: Short Message Service; LBP = low back pain; TS = two-stage subgroups; *p*-values indicate a statistical significant difference between the subgroups for that outcome

For all three types of outcome trajectories (pain intensity, pain frequency, and disability frequency) we found statistically significant differences between the two-stage subgroups and the outcome (*p* < 0.001). Generally, TS1 (mild) patients had the mildest trajectory across SMS outcomes and TS2 (mild, work issues), TS3 (mild, sleep well), TS5 (sleep issues), TS6 (work-related) and TS8 (very recent) patients differed from those in TS1 (mild) by having a slightly higher pain intensity and frequency. The TS7 (nerve root) and TS9 (severe) patients had the most severe pain intensity trajectories but differed from each other as TS7 (nerve root) patients had a higher frequency of days with activity limitation and pain. The TS4 (mild persistent) patients differed from TS9 (severe) patients by having slightly lower pain intensity and fewer days with activity limitation (Fig. 4).

#### Disability

At all three follow-up time points, there was a statistically significant association between the two-stage subgroups and disability (Table 3). Broadly, the TS1 (mild) patients had the lowest disability score at all follow-up time points followed by TS2 (mild, work issues) and TS3 (mild, sleep well) patients. The patients in subgroups TS5 (sleep issues), TS6 (work-related) and TS8 (very recent) all mainly differed to TS2 (mild, work issues) and TS3 (mild, sleep well) patients by having a bigger decrease in disability over time, in part made possible by their having a higher disability score at 2 weeks. The patients in subgroups TS7 (nerve root) and TS9 (severe) had the highest disability scores overall. The TS4 (mild persistent) patients had quite low disability scores and the most consistent score over time (Fig. 3).

Across all outcomes, the general order of the subgroups on outcome severity was TS1 (mild), TS2 (mild, work issues), TS3 (mild, sleep well), TS8 (very recent), TS5 (sleep issues), TS6 (work-related), TS4 (mild persistent), TS9 (severe) and TS7 (nerve root).

### Prognostic capacity

An overview of the prognostic capacity of the subgroupings compared to the prognostic variables used in this study is presented in Table 4.

**Table 4:** An overview of the prognostic capacity of the new subgroupings compared to that of the comparator prognostic variables

Outcome measure	Description of prognostic variable	Prognostic variable	Single-stage and two-stage subgroupings			
			Table 3 (absolute outcomes)	Table 5 (in addition to baseline values)	Table 6 (in addition to recovery belief)	Table 7 (in addition to recovery belief and baseline values)
Pain intensity (Numeric Rating Scale, 0–10)	Existing subgrouping tools	STarT Back Tool	Higher^a^	Higher	Higher	Higher
		Quebec Task Force classification	Higher	Higher	Similar at 3 m, higher at 2 w and 12 m	Similar at 3 m, higher at 2 w and 12 m
	Baseline characteristics	Baseline back pain intensity (0–10 Numeric Rating Scale)	Higher	N/A	Higher	N/A
		Baseline leg pain intensity (0–10 Numeric Rating Scale)	Higher	Similar at 2 w and 12 m, higher at 3 m	Similar at 2 w and 12 m, higher at 3 m	Lower at 2 w and 12 m, higher at 3 m
		Baseline disability (RMDQ-23), (0–100 = highest disability)	Higher	Higher	Higher	Higher
		Recovery belief (0–10 = very likely)	Similar at 2 w and 12 m, lower at 3 m	Similar at 2 w and 12 m, lower at 3 m	N/A	N/A
	Results of first stage of two-stage LCA	All domain-specific patient categorisations	Lower^b^	Lower	Lower	Lower
Disability (23-item Roland Morris Disability Questionnaire, proportional score)	Existing subgrouping tools	STarT Back Tool	Higher	Higher	Higher	Higher
		Quebec Task Force classification	Higher	Higher	Higher	Similar 12 m, higher at 2 w and 3 m
	Baseline characteristics	Baseline back pain intensity (0–10 Numeric Rating Scale)	Higher	Higher	Higher	Higher
		Baseline leg pain intensity (0–10 Numeric Rating Scale)	Higher	Higher	Similar at 12 m, higher at 2 w and 3 m	Lower at 12 m, higher at 2 w and 3 m
		Baseline disability (RMDQ-23), (0–100 = highest disability)	Similar at 2 w, higher at 3 m and 12 m	N/A	Lower at 2 w, similar at 12 m, higher at 3 m	N/A
		Recovery belief (0–10 = very likely)	Similar at 3 m, lower at 12 m, higher at 2 w	Lower 3 m and 12 m, higher at 2 w	N/A	N/A
	Results of first stage of two-stage LCA	All domain-specific patient categorisations	Lower	Lower	Lower	Lower

*m* months, *w* weeks, *N/A* not applicable as the variables were taking into account in the regression model and were therefore not the variable of interest

^a^Higher: if the subgroupings had a higher prognostic capacity than that of the prognostic variable at all three time points, when it was not it is stated

^b^Lower: if the subgroupings had a lower prognostic capacity than that of the prognostic variable at all three time points

#### Prognostic capacity for absolute outcomes

At each follow-up, the prognostic capacity of the single-stage and the two-stage subgroupings was similar on both the outcomes of pain intensity and disability (Table 3). Overall, the prognostic capacity of both subgroupings was better than the SBT and Quebec classifications for pain intensity as well as disability. This was the same, when comparing the subgroupings to the baseline question about disability, except for the prognostic capacity at 2 weeks being no better for the outcome of disability. The single question about the participants’ recovery belief had a higher prognostic capacity than both of the new subgroupings for pain intensity at 3 months and disability at 12 months, a similar prognostic capacity for pain intensity at 2 weeks and 12 months and disability at 3 months, but lower at 2 weeks for disability. The remaining baseline characteristics had a lower prognostic capacity than both of the new subgroupings for both outcomes and at all the time points. Interestingly, the regression model that included the six independent domain-specific patient categorisations (first stage of the two-stage approach) had a distinctly higher prognostic capacity compared to all of the other investigated prognostic models on both outcomes and at all three time points.

#### Prognostic capacity in addition to the baseline values of the outcome measures

As expected, the prognostic capacity ascribed to the subgroups decreased when including the baseline values of the outcomes, and still there was no difference between the two LCA approaches to subgrouping (Table 5). Again, the prognostic capacity for both subgroupings was better compared to SBT and Quebec classifications for pain intensity, as well as for disability. However, in this model the single question about baseline leg pain intensity had similar prognostic capacity to the subgroupings at 2 weeks and 12 months for the outcome of pain intensity, but as in the previous model, a lower prognostic capacity at 3 months and at all time points for the outcome of disability. Similarly to the previous model, both subgroupings had a distinctly higher prognostic capacity at all time points, compared to the baseline question of disability for the outcome of pain intensity. This was the same when compared to the baseline question of pain intensity for the outcome of disability. As before, the single question about the participants’ recovery belief had a somewhat higher prognostic capacity than both of the new subgroupings for pain intensity at 3 months, and a similar prognostic capacity for pain intensity at 2 weeks and 12 months, but a higher prognostic capacity at both 3 and 12 months for the outcome of disability. Again, the regression model that included the six independent domain-specific patient categorisations had a distinctly higher prognostic capacity compared to all of the other investigated prognostic models on both outcomes and at all three time points.

**Table 5:** Prognostic capacity in addition to that of the baseline values of the outcome measures

			Follow-up time points		
Outcome measure	Description of prognostic variable	Prognostic variable	2 weeks	3 months	12 months
			Diff. of. adj. R^2^	Diff. of. adj. R^2^	Diff. of. adj. R^2^
Pain intensity (Numeric Rating Scale, 0–10)	LCA-derived subgroupings	Single-stage subgroups	0.053**	0. 045**	0.055**
		Two-stage subgroups	0.047**	0.052**	0.047**
	Existing subgrouping tools	STarT Back Tool	0.022**	0.006	0.008
		Quebec Task Force classification	0.016*	0.013*	0.007
	Baseline characteristics	Baseline leg pain intensity (0–10 Numeric Rating Scale)	0.047**	0.014*	0.040**
		Baseline disability (RMDQ-23) (0–100 = highest disability)	0.003	−0.000	−0.001
		Recovery belief (0–10 = very likely)	0.060**	0.105**	0.056**
	Results of first stage of two-stage LCA	All domain-specific patient categorisations	0.086**	0.127**	0.135**
Disability (23-item Roland Morris Disability Questionnaire, proportional score)	LCA-derived subgroupings	Single-stage subgroups	0.069**	0.078**	0.059**
		Two-stage subgroups	0.061**	0.074**	0.050**
	Existing subgrouping tools	STarT Back Tool	0.031**	0.040**	0.017*
		Quebec Task Force classification	0.024**	0.042**	0.037**
	Baseline characteristics	Baseline back pain intensity (0–10 Numeric Rating Scale)	−0.011	−0.004	−0.009
		Baseline leg pain intensity (0–10 Numeric Rating Scale)	0.024**	0.034**	0.041**
		Recovery belief (0–10 = very likely)	0.036**	0.115**	0.114**
	Results of first stage of two-stage LCA	All domain-specific patient categorisations	0.086**	0.155**	0.154**

Diff. of. adj. R^2^ = difference in adjusted R^2^ obtained when the prognostic variable was added; LCA = Latent Class Analysis

*P < .05, **P < .001

#### Prognostic capacity in addition to participants’ recovery beliefs

Once again, the prognostic capacity of the new subgroupings was very similar for both outcomes when added to that of participants’ recovery beliefs (Table 6). Also, the prognostic capacity for both subgroupings was better compared to both the SBT and Quebec classifications for pain intensity as well as disability, with the exception that the Quebec classification was similar to both subgroupings for pain intensity at 3 months. Again, both subgroupings at all time points and for both outcomes had a higher prognostic capacity than that of baseline back pain intensity. Also, the single question about baseline leg pain intensity had similar prognostic capacity to that of the subgroupings on the outcomes of pain intensity at 2 weeks and 12 months, but also for disability at 12 months, and a lower prognostic capacity at other time points. For the outcome of pain intensity, the prognostic capacity of the subgroupings was better than that of the baseline disability question at all time points, but for the outcome of disability, the prognostic capacity of the subgroupings was better at 3 months, similar at 12 months and lower at 2 weeks. As before, the regression model including the six independent domain-specific patient categorisations (first stage of the two-stage approach) had a distinctly higher prognostic capacity than all the other investigated prognostic models on both outcomes and at all three time points.

**Table 6:** Prognostic capacity in addition to that of the participants’ recovery beliefs

			Follow-up time points		
Outcome measure	Description of prognostic variable	Prognostic variable	2 weeks	3 months	12 months
			Diff. of. adj. R^2^	Diff. of. adj. R^2^	Diff. of. adj. R^2^
Pain intensity (Numeric Rating Scale, 0–10)	LCA-derived subgroupings	Single-stage subgroups	0.041**	0.013*	0.033**
		Two-stage subgroups	0.039**	0.015*	0.021*
	Existing subgrouping tools	STarT Back Tool	0.023**	0.004	0.008*
		Quebec Task Force classification	0.007	0.012*	0.005
	Baseline characteristics	Baseline back pain intensity (0–10 Numeric Rating Scale)	0.024**	0.002*	−0.003*
		Baseline leg pain intensity (0–10 Numeric Rating Scale)	0.049**	0.004*	0.027**
		Baseline disability (RMDQ-23) (0–100 = highest disability)	0.026**	0.001	0.007*
	Results of first stage of two-stage LCA	All domain-specific patient categorisations	0.052**	0.047**	0.074**
Disability (23-item Roland Morris Disability Questionnaire, proportional score)	LCA-derived subgroupings	Single-stage subgroups	0.183**	0.084**	0.051**
		Two-stage subgroups	0.174**	0.084**	0.044**
	Existing subgrouping tools	STarT Back Tool	0.105**	0.059**	0.023**
		Quebec Task Force classification	0.033**	0.037**	0.029*
	Baseline characteristics	Baseline back pain intensity (0–10 Numeric Rating Scale)	0.057**	0.013*	0.009*
		Baseline leg pain intensity (0–10 Numeric Rating Scale)	0.052**	0.036**	0.048**
		Baseline disability (RMDQ-23) (0–100 = highest disability)	0.199**	0.069**	0.049**
	Results of first stage of two-stage LCA	All domain-specific patient categorisations	0.232**	0.123**	0.101**

Diff. of. adj. R^2^ = difference in adjusted R^2^ obtained when the prognostic variable was added; *LCA* Latent Class Analysis

*P < .05

**P < .001

#### Prognostic capacity in addition to the baseline values of the outcomes and participants’ recovery beliefs

Here also, there was no difference in prognostic capacity of the new subgroupings for the outcomes of pain intensity and disability when taking into account both the baseline values of these outcomes and participants’ recovery beliefs (Table 7). Again, a better prognostic capacity were seen for both subgroupings when compared to that of SBT and Quebec classification for pain intensity as well as disability, with the exception that the Quebec classification was similar to both subgroupings for pain intensity at 3 months but, in this case, also for disability at 12 months. As before, both subgroupings and at all time points, had a distinctly higher prognostic capacity when compared to the baseline question of disability for the outcome of pain intensity, and compared to the baseline question of pain intensity for the outcome of disability. In this model, the baseline leg pain intensity question had a slightly higher prognostic capacity compared to the subgroupings for pain intensity at 2 weeks and 12 months, and for disability at 12 months, but as before, a lower prognostic capacity at other time points. Once again, the regression model that included the six independent domain-specific patient categorisations (first stage of the two-stage approach) had a distinctly higher prognostic capacity compared to all the other investigated prognostic models on both outcomes and at all three time points.

**Table 7:** Prognostic capacity in addition to that of the baseline values of the outcomes measures and participants’ recovery beliefs

			Follow-up time points		
Outcome measure	Description of prognostic variable	Prognostic variable	2 weeks	3 months	12 months
			Diff. of. adj. R^2^	Diff. of. adj. R^2^	Diff. of. adj. R^2^
Pain intensity (Numeric Rating Scale, 0–10)	LCA-derived subgroupings	Single-stage subgroups	0.028*	0.014*	0.026**
		Two-stage subgroups	0.022*	0.013*	0.019*
	Existing subgrouping tools	STarT Back Tool	0.014*	0.001	0.003
		Quebec Task Force classification	0.006	0.010*	0.005
	Baseline characteristics	Baseline leg pain intensity (0–10 Numeric Rating Scale)	0.038**	0.007*	0.033**
		Baseline disability (RMDQ-23) (0–100 = highest disability)	0.006	−0.001	0.000
	Results of first stage of two-stage LCA	All domain-specific patient categorisations	0.042**	0.049**	0.077**
Disability (23-item Roland Morris Disability Questionnaire, proportional score)	LCA-derived subgroupings	Single-stage subgroups	0.047**	0.041**	0.024*
		Two-stage subgroups	0.041**	0.035*	0.016
	Existing subgrouping tools	STarT Back Tool	0.020**	0.020**	0.003
		Quebec Task Force classification	0.016**	0.027*	0.026*
	Baseline characteristics	Baseline back pain intensity (0–10 Numeric Rating Scale)	−0.009	−0.003	−0.007
		Baseline leg pain intensity (0–10 Numeric Rating Scale)	0.032**	0.028**	0.042**
	Results of first stage of two-stage LCA	All domain-specific patient categorisations	0.054**	0.067**	0.064**

Diff. of. adj. R^2^ = difference in adjusted R^2^ obtained when the prognostic variable was added; *LCA*  Latent Class Analysis

*P < .05

**P < .001

**Fig. 5:**
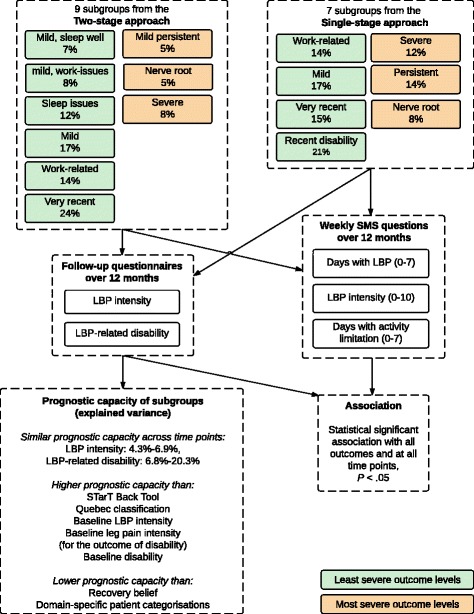
Overview of method and results. LBP = low back pain

An overview of the process and final results are shown in Fig. 5.

## Discussion

This study investigated the prognostic capacity of two novel LCA-derived subgroupings of patients with LBP. Neither of these LCA approaches were judged to have a better prognostic capacity then the other, as the single-stage and the two-stage subgroupings were similarly associated with the questionnaire-based outcomes of pain intensity and disability at 2 weeks, 3 and 12 months, and were similarly associated with the weekly SMS-collected outcomes of pain intensity, pain frequency and disability over 12 months. Their prognostic capacity was similar to, or somewhat better, than the two existing subgrouping tools that were included for comparison in this study for the outcome measures of pain intensity and disability. Their prognostic capacity was also similar to, or somewhat better than, the prognostic capacity of baseline LBP intensity and disability. These two novel subgroupings generally had a higher prognostic capacity than that of the baseline leg pain intensity for the outcome of disability, but not consistently so for the outcome of pain intensity. Compared to participants’ recovery beliefs, the subgroupings had a lower prognostic capacity for the outcome of pain intensity at 3 months and for the outcome of disability at 12 months, and similar prognostic capacity for the outcome pain intensity at 2 weeks and 12 months and for the outcome of disability at 3 months. These novel subgroupings also had a lower prognostic capacity than that of the domain-specific patient categorisations formed in the first stage of the two-stage LCA approach. The prognostic capacity of these two novel subgroupings was limited in size for the outcome of pain intensity (adjusted R^2^ ranging from 4.3% to 6.9%), but more substantial for the outcome of disability (6.8% to 20.3%), and highest at the first follow-up time point. The subgroupings did not provide substantial additional prognostic information when added to regression models that included the baseline values of participants’ recovery beliefs and either pain or disability.

### Association between the subgroups and outcomes

On both the questionnaire and SMS based outcome measures, the subgroups generally showed the same order of outcome severity and differed statistically on all outcome measures, albeit that the differences between some of the subgroups were small, especially at 3 and 12 months.

#### Characteristics of subgroups with the least and most severe outcome levels

To understand the relationship between baseline characteristics and outcomes, it can be helpful to consider these subgroups relative to the least and most severe outcome levels. Subgroups showing the lowest severity level over time were either mildly affected at baseline regardless of the duration of their LBP or were severely affected at baseline but with recent onset. In addition, they differed on work-issues, sleep-issues, degree of activity limitation and sacroiliac joint pain. However, one exception was the subgroup TS4 (mild persistent) whose members were also mildly affected at baseline, but differed from the others as they had a high severity level over time. Patients in this subgroup, and the subgroups that were severely affected at baseline, differed qualitatively on the degree of nerve root involvement, psychological issues and persistence of their LBP. This aligns with previous evidence for markers of a poor prognosis [17, 40, 48]. It also suggests a need to differentiate between potential causes of poor outcome rather than simply summing prognostic factors into one prognostic sum score (‘vote counting’). For example, the prognostic capacity of leg pain has been questioned, as this might be explained by other concurrent baseline characteristics, such as differences in pain duration, LBP severity, demographic and psychological characteristics [49]. However, another explanation could be that the prognostic capacity of leg pain depends on its relationship with other baseline characteristics. In our study, the subgroups with the most severe outcome level also included the largest proportion of patients with leg pain. However, while for the subgroups characterised by nerve root involvement (SS4 and TS7) leg pain seemed to inform the subgrouping, in contrast, in the other subgroups characterised by persistent LBP (SS6 and TS4) and severe LBP (SS7 and TS9), it seemed to have less impact on the subgrouping. Therefore, other characteristics, or potentially a group of characteristics, seem to have informed the subgrouping. So, despite similar outcome measures for the most severe subgroups, these qualitative differences may provide pointers towards ways in which these subgroups could be managed and treated differently to obtain a greater improvement. This deserves further investigation.

### Prognostic capacity

The prognostic capacity of the two new subgroupings were similar across time points and outcome measures, and therefore neither of these subgroupings was more strongly or consistently associated with outcomes than the other. However, the prognostic capacity of the new subgroupings was somewhat better than that of the two existing subgrouping tools used for comparison and therefore, there seems to be potentially important information within these new subgroups. This may be predictable, given that the new subgroupings were based on the same baseline characteristics covered by the existing subgrouping tools, plus additional baseline information. However, this indicates that additional prognostic information may be derived from clusters of the variables that are traditionally measured at an initial consultation for LBP.

Still, it remains unclear how we can best extract and use this information. In particular, the domain-specific patient categorisations from the first stage of the two-stage approach showed a quite large prognostic capacity when entered directly into regression models. This indicates that the approach to aggregate information into a simple subgrouping – as done by the two-stage approach – is suboptimal with respect to the use of the available information. However, the prediction rule based on using the domain-specific categorisations as input would not currently be directly applicable in practice. It would require a determination of each patient’s assignment to a subgroup within each domain and then to build a summary score model involving overall 37 weights. Hence, there remains a task to find better and simpler ways to aggregate these domain specific categorisations.

### Strengths and limitations

One strength of this study was the use of multiple and frequently used LBP outcome measures to test novel LCA-derived subgroupings. Another strength was that the LCA-derived subgroupings were formed in a large sample of LBP patients and these subgroups were selected not only on statistical criteria but also on their qualitative differences. This allowed identification of otherwise unrecognised relationships between baseline characteristics and was used to incorporate more of the clinical complexity of LBP into this prognostic study. The extent to which the results of such complex subgroupings can be generalised to other patient populations is unknown and this should be investigated.

Comparing the prognostic capacity of our novel subgrouping approaches to two other subgrouping tools (one based on patient-reported questionnaire responses and one that included clinical examination findings), and other known predictors, also strengthened our study. However, as there is evidence that the predictive ability of the SBT is stronger in populations with LBP of more than 2 weeks duration [50], which was only 36% of this cohort, it is unknown if the relative performance of these novel subgroupings would be different in other settings.

In addition, we recognise that neither the Quebec classification nor the SBT are primarily designed to be prognostic tools. However, the Quebec classifications, especially the characteristics of pain localisation and neurological signs have been shown to influence prognosis and therefore, the use of the Quebec classification as a comparator was considered reasonable [18, 51]. The SBT was developed to stratify treatment. If such stratified treatment had been applied by the chiropractors, either consciously or subconsciously, the prognostic value of the SBT could have been reduced due to appropriate treatment matching resulting in better patient outcomes in all risk groups. The same issue may relate to the Quebec classification and the LCA derived classifications if that somehow guided treatment decisions. Therefore, the study might have been strengthened had adjustment for any differences in the type of treatment and the number of treatment sessions been performed as the specific characteristics of each subgroup may have had an influence on treatment decisions. Unfortunately, detailed treatment information was not obtained in the original data collection. As a result, we cannot rule out that the observed outcome differences between subgroups are confounded with treatment differences between subgroups, although we know the participants received a quite homogeneous treatment package by the chiropractors consisting of health information, manual treatment and exercises. As always, less loss to follow-up would have strengthened the study and reduced any potential responder bias.

In evaluating the subgroupings derived from the LCA approaches, we ignored that there may have been potential misclassification of some subjects, as we relied purely on the highest posterior probability. Alternatively, we may have used the posterior probabilities themselves as input to the regression models (i.e. considered their prognostic capacity) or may have used approaches to correct for bias due to misclassification (BCH, [52, 53]). However, this would not directly address the question about the prognostic capacity of the subgroupings themselves.

In this paper, we did not address the clinically feasibility of the subgroupings, e.g. whether they can be identified in practice based only on the verbal description or whether they can be implemented by using algorithmic support. Taking the rather low prognostic capacity into account, there is currently no merit for investigating this aspect. However, the promising results when using the domain specific categorisations and the role of single variables like the patient’s recovery beliefs may pave a way to obtain clinically feasible subgroupings or scoring rules in the future based on our data.

## Conclusions

Subgroups can have clinical significance if they predict better response to more targeted treatment and/or if they allow more precise estimates of prognosis. In this study, we only investigated the prognostic implications of these novel subgroups and found a relatively small amount of added prognostic capacity compared to simpler subgrouping tools. Consequently, we do not currently see direct implications of these LCA-derived subgroupings for clinical practice, in particular as their complexity suggests that direct clinical application would require software support. However, if these novel subgroups were further investigated and found to respond better to treatment that targets subgroup-specific modifiable factors, then a clinical implication of these subgroups may appear. Our current prognostic findings do not justify developing these new subgroupings into clinical prediction rules, especially when taking into account the comprehensive and time-consuming baseline questionnaires that formed the subgroupings. However, further research could aim at identifying the distinct characteristics that carried additional prognostic capacity in order to improve existing subgrouping tools and also investigate if distinctive aspects of these subgroups might be suitable for targeted treatment. Furthermore, the characteristics that differentiated these subgroups could be investigated in a causal model framework to determine whether they suggest the existence of different casual pathways.

In addition, the lower prognostic capacity of the two-stage subgrouping, compared to using domain-specific patient categorisations, indicates that there is an information loss going from the first to the second stage of the two-stage LCA approach. This warrants further methodological investigation from both a statistical and a conceptual perspective.

## Additional file

Additional file 1: Table A1. Presentation of Latent Class Analysis derived domain-specific patient categories for each of the six health domains. **Table A2a.** Baseline characteristics of Latent Class Analysis derived single-stage subgroups. **Table A2b.** Baseline characteristics of Latent Class Analysis derived two-stage subgroups. **Table A3.** Test of differences between responders and non-responders on the follow-up questionnaires. **Table A4.** Test of differences between compliant responders and non-compliant responders on the SMS questions over 12 months. (DOCX 55 kb)

## Abbreviations

LBP: Low Back Pain
LCA: Latent Class Analysis
RMDQ: Roland-Morris Disability Questionnaire
SBT: STarT Back Tool
SMS: Short Message Service
SS: Single-stage patient Subgroup
TS: Two-stage patient Subgroup
