# Supplementary material for: Latent class analysis derived subgroups of low back pain patients – do they have prognostic capacity?
Source: BMC Musculoskelet Disord. 2017 Aug 9;18:345. doi: 10.1186/s12891-017-1708-9 (PMC5551030; doi:10.1186/s12891-017-1708-9)
Supplement: Additional file 1: Table A1. — Presentation of Latent Class Analysis derived domain-specific patient categories for each of the six health domains. Table A2a. Baseline characteristics of Latent Class Analysis derived single-stage subgroups. Table A2b. Baseline characteristics of Latent Class Analysis derived two-stage subgroups. Table A3. Test of differences between responders and non-responders on the follow-up questionnaires. Table A4. Test of differences between compliant responders and non-compliant responders on the SMS questions over 12 months. (DOCX 55 kb) [file 12891_2017_1708_MOESM1_ESM.docx]

# Additional file 1

*Manuscript: Latent Class Analysis derived subgroups of low back pain patients – do they have prognostic capacity?
Nielsen AM, Hestbaek L, Vach W, Kent P, Kongsted A*

## Table A1. Presentation of Latent Class Analysis derived domain-specific patient categories for each of the six health domains

| **Health domain and category description** | | | | | **Prevalence, N=928** |
| --- | --- | --- | --- | --- | --- |
| **Activity domain** | | | | | |
| Act. 1: | Very high degree of disability (transfer/climbing/bending/turning/walking/dressing/household/work) | | | | 254 (27%) |
| Act. 2: | Very high degree of disability, but no walking distance limitation | | | | 223 (24%) |
| Act. 3: | Very low degree of disability | | | | 137 (15%) |
| Act. 4: | Low degree of disability, but dressing problems | | | | 105 (11%) |
| Act. 5: | Moderate degree of disability, no walking distance limitations | | | | 79 (9%) |
| Act. 6: | Low degree of disability, but difficulties in household duties | | | | 81 (9%) |
| Act. 7: | Moderate degree of disability, high degree of walking limitations (speed and distance) | | | | 49 (5%) |
| **Contextual factors domain** | | | | | |
| Context 1: | | | Healthy males working full‐time or self‐employed | | 302 (33%) |
| Context 2: | | | Healthy females working full‐time or part‐time | | 191 (21%) |
| Context 3: | | | Females with comorbidity, working full‐time or part‐time | | 152 (16%) |
| Context 4: | | | Males with comorbidity, working full‐time or self‐employed | | 148 (16%) |
| Context 5: | | | Healthy patients in their 30s, higher body mass index, working full-time, students or unemployed | | 54 (6%) |
| Context 6: | | | Healthy students (approx. 25 years of age), lower body mass index, fewer with health insurance | | 40 (4%) |
| Context 7: | | | Retired patients (or working part‐time) with comorbidity, fewer with health insurance | | 41 (4%) |
| **Pain domain** | | | | | |
| Pain 1: | Recent LBP with high degree of back pain severity | | | | 252 (27%) |
| Pain 2: | Recent LBP with high degree of back pain severity and moderate degree of leg pain severity | | | | 203 (22%) |
| Pain 3: | Recent LBP with low degree of back pain severity | | | | 160 (17%) |
| Pain 4: | Persistent LBP, high degree of back and leg pain severity | | | | 98 (11%) |
| Pain 5: | Recent LBP with moderate degree of back pain severity, moderate degree of leg pain severity | | | | 89 (10%) |
| Pain 6: | Persistent LBP, moderate degree of back pain severity and low degree of leg pain severity | | | | 84 (9%) |
| Pain 7: | Recent LBP, moderate degree of LBP severity, moderate to high degree of leg pain severity, non-dominating LBP | | | | 42 (5%) |
| **Participation domain** | | | | | |
| Part. 1: | Very few work and social participation limitations, low degree of physical workload | | | | 348 (38%) |
| Part. 2: | Very few social participation limitations, unsure if work aggravated/makes worse, whichever degree of physical workload | | | | 154 (17%) |
| Part. 3: | Very few social participation limitations, work is too heavy, aggravated/makes worse, pain caused by or at work, high degree of physical workload | | | | 128 (14%) |
| Part. 4: | High degree of social participation limitations, but low degree of work limitations whichever degree of physical workload | | | | 93 (10%) |
| Part. 5: | Moderate degree of social participation limitations, work aggravated/makes pain worse, very low degree of physical workload | | | | 77 (8%) |
| Part. 6: | Low degree of social participation limitations, pain caused by work, none with very low degree of physical workload | | | | 70 (8%) |
| Part. 7: | High degree of social participation limitations, work is too heavy, aggravated/makes worse, pain caused by work, high degree of physical workload | | | | 58 (6%) |
| **Physical impairment domain** | | | | | |
| Phy.imp. 1: | | | | LBP on flexion and extension, no leg pain | 264 (28%) |
| Phy.imp. 2: | | | | LBP on flexion, extension and side glide, no leg pain, sacroiliac joint pain, trigger points and painful buttock/leg muscles | 180 (19%) |
| Phy.imp. 3: | | | | LBP on flexion, extension and side glide, no leg pain, diagnosis: reducible disc | 147 (16%) |
| Phy.imp. 4: | | | | LBP on flexion, extension and slide glide, no leg pain, diagnosis: partly reducible disc | 138 (15%) |
| Phy.imp. 5: | | | | LBP on active range of motion in all directions, painful back muscles | 126 (14%) |
| Phy.imp. 6: | | | | Leg pain on flexion, extension and side glide, neurological signs, trigger points and painful buttock/leg muscles | 73 (8%) |
| **Psychology domain** | | | | | |
| Psych. 1: | | Treatment efficacy believers with low degree of depressive mood | | | 199 (21%) |
| Psych. 2: | | Pain-related concerns, moderate degree of depressive mood | | | 154 (17%) |
| Psych. 3: | | Uncomplicated psychological profile | | | 132 (14%) |
| Psych. 4: | | Sleep well, low degree of depressive mood | | | 119 (13%) |
| Psych. 5: | | Treatment efficacy believers with sleep issues and moderate degree of depressive mood | | | 122 (13%) |
| Psych. 6: | | Sleep issues, low degree of pain-related concerns | | | 99 (11%) |
| Psych. 7: | | A complicated psychological profile | | | 55 (6%) |
| Psych. 8: | | Pain-related concerns, low degree of depressive mood | | | 48 (5%) |
| *LBP = low back pain.* | | | | | |

## Table A2a. Baseline characteristics of Latent Class Analysis derived single-stage subgroups

| **Subgroup**  **Baseline characteristic** | **SS1**  **(mild)**  **N=154** | **SS2**  **(recent disability)**  **N=192** | **SS3**  **(work-related)**  **N=130** | **SS4**  **(nerve root)**  **N=75** | **SS5**  **(very recent)**  **N=136** | **SS6**  **(persistent)**  **N=132** | **SS7**  **(severe)**  **N=109** | ***P* value*** |
| --- | --- | --- | --- | --- | --- | --- | --- | --- |
| Males, N (%) | 90 (58) | 111 (58) | 99 (76) | 22 (29) | 78 (57) | 64 (48) | 46 (42) | < .001^¤^ |
| Age, median (interquartile range, years) | 41  (33-49) | 48  (39-56) | 38  (29-51) | 49  (38-57) | 43  (35-53) | 39  (30-50) | 44  (35-52) | < .001^#^ |
| Highest achieved education, N (%) |  |  |  |  |  |  |  | < .001^#^ |
| No qualification | 8 (5) | 6 (3) | 21 (16) | 4 (5) | 12 (9) | 14 (11) | 16 (15) |  |
| Vocational training | 27 (18) | 37 (19) | 58 (45) | 14 (19) | 41 (30) | 26 (20) | 33 (30) |  |
| Higher education < 3 years | 25 (16) | 29 (15) | 18 (14) | 15 (20) | 20 (15) | 16 (12) | 19 (17) |  |
| Higher education 3-4 years | 52 (34) | 84 (44) | 22 (17) | 31 (41) | 46 (34) | 47 (36) | 29 (27) |  |
| Higher education >4 years | 38 (25) | 35 (18) | 9 (7) | 7 (9) | 13 (10) | 26 (20) | 8 (7) |  |
| Missing | 4 (3) | 1 (1) | 2 (2) | 4 (5) | 4 (3) | 3 (2) | 4 (4) |  |
| Episode duration, N (%) |  |  |  |  |  |  |  | < .001^#^ |
| 0-2 weeks | 63 (41) | 140 (73) | 72 (55) | 41 (55) | 127 (93) | 53 (40) | 75 (69) |  |
| 2-4 weeks | 32 (21) | 20 (10) | 21 (16) | 8 (11) | 2 (1) | 27 (20) | 13 (12) |  |
| 1-3 months | 24 (16) | 18 (9) | 13 (10) | 14 (19) | 1 (1) | 17 (13) | 8 (7) |  |
| >3 months | 33 (21) | 13 (7) | 23 (18) | 10 (13) | 1 (1) | 32 (24) | 9 (8) |  |
| Missing | 2 (1) | 1 (1) | 1 (1) | 2 (3) | 5 (4) | 3 (2) | 4 (4) |  |
| Back pain intensity (0-10 Numeric Rating Scale), median (interquartile range) | 4 (3-6) | 7 (5-8) | 7 (5-8) | 7 (6-8) | 8 (7-9) | 7 (6-8) | 8 (7-9) | < .001^#^ |
| Missing, N(%) | 5 (3) | 3 (2) | 1 (1) | 4 (5) | 5 (4) | 3 (2) | 4 (4) |  |
| Any leg pain, N(%) | 61 (40) | 102 (53) | 79 (61) | 75 (100) | 65 (48) | 89 (67) | 85 (78) | < .001^#^ |
| Leg pain intensity (0-10 Numeric Rating Scale), median (interquartile range) | 0 (0-2) | 1 (0-3) | 1.5 (0-4) | 6 (5-8) | 0 (0-2) | 2 (0-4) | 4 (1.5-6) |  |
| Missing, N(%) | 6 (4) | 11 (6) | 4 (3) | 2 (3) | 10 (7) | 5 (4) | 5 (5) |  |
| STarT Back Tool score, N (%) |  |  |  |  |  |  |  | < .001^#^ |
| Low risk | 143 (93) | 127 (66) | 88 (68) | 34 (45) | 63 (46) | 38 (29) | 4 (4) |  |
| Medium risk | 10 (6) | 61 (32) | 36 (28) | 38 (51) | 68 (50) | 73 (55) | 65 (60) |  |
| High risk | 0 (0) | 4 (2) | 4 (3) | 2 (3) | 4 (3) | 20 (15) | 38 (35) |  |
| Missing | 1 (1) | 0 (0) | 2 (2) | 1 (1) | 1 (1) | 1 (1) | 2 (2) |  |
| Quebec classification, N(%) |  |  |  |  |  |  |  | < .001^¤^ |
| Local LBP only | 122 (79) | 144 (75) | 94 (72) | 36 (48) | 108 (79) | 93 (70) | 48 (44) |  |
| LBP + leg pain above the knee | 19 (12) | 42 (22) | 30 (23) | 0 (0) | 21 (15) | 32 (24) | 38 (35) |  |
| LBP + leg pain below the knee | 9 (6) | 2 (1) | 5 (4) | 27 (36) | 4 (3) | 7 (5) | 15 (14) |  |
| LBP + leg pain and neurological signs | 1 (1) | 0 (0) | 0 (0) | 12 (16) | 1 (1) | 0 (0) | 6 (6) |  |
| Missing | 3 (2) | 4 (2) | 1 (1) | 0 (0) | 2 (1) | 0 (0) | 2 (2) |  |
| Roland-Morris Disability Questionnaire proportional sum score (0-100=highest disability), median (interquartile range) | 17  (9-26) | 52  (43-65) | 39  (28-48) | 57  (39-70) | 78  (70-82) | 56  (43-65) | 78  (73-87) | < .001^#^ |
| Missing, N(%) | 2 (1) | 0 (0) | 2 (2) | 2 (3) | 5 (4) | 2 (2) | 1 (1) |  |
| Recovery belief (0-10=very likely), median (interquartile range) | 10 (8-10) | 10 (8-10) | 8.5 (5-10) | 9 (5-10) | 10 (9-10) | 8 (5-10) | 8 (5-10) | < .001^#^ |
| Missing, N(%) | 1 (1) | 1 (1) | 4 (3) | 1 (1) | 2 (1) | 2 (2) | 2 (2) |  |
| *Test of differences between the subgroups on the baseline characteristics, missing values were excluded when calculating the *P* values  ^¤^χ^2^ test  ^#^Kruskal-Wallis equality-of-populations rank test | | | | | | | | |

## Table A2b. Baseline characteristics of Latent Class Analysis derived two-stage subgroups

| **Subgroup**  **Baseline characteristic** | **TS1**  **(mild)**  **N=161** | **TS2**  **(mild, work issues)**  **N=74** | **TS3**  **(mild, sleep well)**  **N=69** | **TS4**  **(persistent)**  **N=45** | **TS5**  **(sleep issues)**  **N=113** | **TS6**  **(work-related)**  **N=127** | **TS7**  **(nerve root)**  **N=49** | **TS8**  **(very recent)**  **N=219** | **TS9**  **(severe)**  **N=71** | ***P* value*** |
| --- | --- | --- | --- | --- | --- | --- | --- | --- | --- | --- |
| Males, N (%) | 118 (73) | 62 (84) | 30 (43) | 1 (2) | 36 (32) | 110 (87) | 25 (51) | 106 (48) | 22 (31) | < .001^¤^ |
| Age, median (interquartile range, years) | 45  (36-54) | 41  (28-48) | 41  (31-52) | 44  (34-53) | 43  (34-52) | 45  (36-56) | 46  (39-54) | 44  (35-54) | 37  (29-48) | < .001^#^ |
| Highest achieved education, N (%) |  |  |  |  |  |  |  |  |  | < .001^#^ |
| No qualification | 5 (3) | 8 (11) | 8 (12) | 1 (2) | 6 (5) | 14 (11) | 4 (8) | 19 (9) | 16 (23) |  |
| Vocational training | 29 (18) | 17 (23) | 16 (23) | 9 (20) | 21 (19) | 51 (40) | 13 (27) | 58 (26) | 22 (31) |  |
| Higher education < 3 years | 30 (19) | 12 (16) | 10 (14) | 6 (13) | 12 (11) | 14 (11) | 9 (18) | 40 (18) | 9 (13) |  |
| Higher education 3-4 years | 54 (34) | 21 (28) | 30 (43) | 27 (60) | 47 (42) | 32 (25) | 17 (35) | 67 (31) | 16 (23) |  |
| Higher education >4 years | 40 (25) | 15 (20) | 5 (7) | 1 (2) | 26 (23) | 12 (9) | 3 (6) | 27 (12) | 7 (10) |  |
| Missing | 3 (2) | 1 (1) | 0 (0) | 1 (2) | 1 (1) | 4 (3) | 3 (6) | 8 (4) | 1 (1) |  |
| Episode duration, N (%) |  |  |  |  |  |  |  |  |  | < .001^#^ |
| 0-2 weeks | 103 (64) | 33 (45) | 38 (55) | 8 (18) | 58 (51) | 83 (65) | 27 (55) | 193 (88) | 28 (39) |  |
| 2-4 weeks | 26 (16) | 15 (20) | 13 (19) | 3 (7) | 18 (16) | 16 (13) | 8 (16) | 11 (5) | 13 (18) |  |
| 1-3 months | 19 (12) | 11 (15) | 7 (10) | 11 (24) | 21 (19) | 11 (9) | 5 (10) | 5 (2) | 5 (7) |  |
| >3 months | 11 (7) | 15 (20) | 11 (16) | 22 (49) | 15 (13) | 15 (12) | 7 (14) | 2 (1) | 23 (32) |  |
| Missing | 2 (1) | 0 (0) | 0 (0) | 1 (2) | 1 (1) | 2 (2) | 2 (4) | 8 (4) | 2 (3) |  |
| Back pain intensity (0-10 Numeric Rating Scale), median (interquartile range) | 6 (4-7) | 5 (3-7) | 5 (3.5-7) | 6 (4-7) | 7 (6-8) | 7 (5-8) | 8 (6-8) | 8 (7-9) | 8 (6-8) | < .001^#^ |
| Missing, N(%) | 5 (3) | 0 (0) | 1 (1) | 2 (4) | 1 (1) | 4 (3) | 3 (6) | 8 (4) | 1 (1) |  |
| Any leg pain, N(%) | 66 (41) | 34 (46) | 30 (43) | 31 (69) | 95 (84) | 61 (48) | 48 (98) | 132 (60) | 59 (83) | < .001^#^ |
| Leg pain intensity (0-10 Numeric Rating Scale), median (interquartile range) | 0 (0-2) | 0 (0-2) | 0 (0-2) | 2.5 (0-5) | 4 (2-6) | 0 (0-3) | 6 (5-8) | 2 (0-4) | 3 (1-6) |  |
| Missing, N(%) | 7 (4) | 1 (1) | 3 (4) | 1 (2) | 1 (1) | 11 (9) | 2 (4) | 16 (7) | 1 (1) |  |
| STarT Back Tool score, N (%) |  |  |  |  |  |  |  |  |  | < .001^#^ |
| Low risk | 137 (85) | 61 (82) | 60 (87) | 32 (71) | 50 (44) | 66 (52) | 8 (16) | 69 (32) | 14 (20) |  |
| Medium risk | 22 (14) | 11 (15) | 9 (13) | 10 (22) | 54 (48) | 53 (42) | 31 (63) | 124 (57) | 37 (52) |  |
| High risk | 2 (1) | 1 (1) | 0 (0) | 2 (4) | 9 (8) | 7 (6) | 9 (18) | 24 (11) | 18 (25) |  |
| Missing | 0 (0) | 1 (1) | 0 (0) | 1 (2) | 0 (0) | 1 (1) | 1 (2) | 2 (1) | 2 (3) |  |
| Quebec classification, N(%) |  |  |  |  |  |  |  |  |  | < .001^¤^ |
| Local LBP only | 123 (76) | 61 (82) | 51 (74) | 22 (49) | 60 (53) | 88 (69) | 2 (4) | 160 (73) | 42 (59) |  |
| LBP + leg pain above the knee | 29 (18) | 12 (16) | 12 (17) | 11 (24) | 42 (37) | 28 (22) | 17 (35) | 44 (20) | 23 (32) |  |
| LBP + leg pain below the knee | 7 (4) | 1 (1) | 5 (7) | 8 (18) | 8 (7) | 6 (5) | 17 (35) | 12 (5) | 5 (7) |  |
| LBP + leg pain and neurological signs | 1 (1) | 0 (0) | 0 (0) | 2 (4) | 2 (1) | 1 (1) | 13 (27) | 0 (0) | 1 (1) |  |
| Missing | 1 (1) | 0 (0) | 1 (1) | 2 (4) | 1 (1) | 4 (3) | 0 (0) | 3 (1) | 0 (0) |  |
| Roland-Morris Disability Questionnaire proportional sum score (0-100=highest disability), median (interquartile range) | 35  (22-48) | 22  (13-35) | 26  (17-39) | 26  (13-43) | 57  (43-65) | 52  (39-65) | 74  (65-78) | 78  (70-83) | 61  (48-70) | < .001^#^ |
| Missing, N(%) | 3 (2) | 0 (0) | 1 (1) | 0 (0) | 2 (2) | 1 (1) | 1 (2) | 4 (2) | 2 (3) |  |
| Recovery belief (0-10=very likely), median (interquartile range) | 10  (9-10) | 8 (5-10) | 9 (5-10) | 7 (3-10) | 9 (7-10) | 9 (6-10) | 8 (5-10) | 10  (8-10) | 7 (5-9) | < .001^#^ |
| Missing, N(%) | 0 (0) | 1 (1) | 0 (0) | 2 (4) | 0 (0) | 4 (3) | 1 (2) | 3 (1) | 2 (3) |  |
| *Test of differences between the subgroups on the baseline characteristics, missing values were excluded when calculating the *P* values  ^¤^χ^2^ test  ^#^Kruskal-Wallis equality-of-populations rank test | | | | | | | | | | |

## Table A3. Test of differences between responders and non-responders on the follow-up questionnaires

| **Outcome variables**  **Baseline characteristics** | **Pain intensity**  **(responders/non-responders to follow-ups)** | | | **Roland-Morris disability**  **(responders/non-responders to follow-ups)** | | |
| --- | --- | --- | --- | --- | --- | --- |
|  | **2 weeks** | **3 months** | **12 months** | **2 weeks** | **3 months** | **12 months** |
| Missing values, N* | 310 (33%) | 199 (21%) | 246 (27%) | 155 (17%) | 196 (21%) | 245 (26%) |
| Males (*P* value) | 53% / 59%  (.10)^¤^ | 53% / 61%  **(.04)** | 53% / 61%  **(.03)** | 54% / 60%  (.17) | 53% / 62%  **(.03)** | 53% / 62%  **(.01)** |
| Age, median (*P* value) | 45 / 41  **(< .001)**^#^ | 45 / 39  **(< .001)** | 45 / 38  **(< .001)** | 45 / 38  **(< .001)** | 45 / 39  **(< .001)** | 45 / 39  **(< .001)** |
| STarT Back Tool, *P* value****** | .83^#^ | **.03** | .19 | .25 | .05 | .13 |
| Low risk group | 54% / 54% | 56% / 47% | 55% / 50% | 55% / 49% | 56% / 47% | 56% / 49% |
| Medium risk group | 38% / 38% | 36% / 45% | 37% / 42% | 37% / 43% | 37% / 44% | 37% / 43% |
| High risk group | 8% / 8% | 8% / 9% | 8% / 8% | 8% / 8% | 8% / 8% | 8% / 8% |
| Quebec classification  (*P* value)****** | .98^¤^ | .88 | .92 | .40 | .94 | .97 |
| Local LBP | 66% / 67% | 67% / 65% | 66% / 67% | 67% / 62% | 67% / 65% | 67% / 66% |
| LBP + leg pain above the  knee | 24% / 23% | 23% / 26% | 24% / 22% | 23% / 25% | 23% / 25% | 24% / 24% |
| LBP + leg pain below the  knee | 8% / 8% | 8% / 7% | 7% / 8% | 7% / 10% | 8% / 7% | 7% / 8% |
| LBP + leg pain and  neurological signs | 2% / 2% | 2% / 3% | 2% / 2% | 2% / 3% | 2% / 3% | 2% / 2% |
| Back pain intensity (0-10 Numeric Rating Scale), median (*P* value) | 7 / 7  (.82)^#^ | 7 / 7  (.41) | 7 / 7  (.36) | 7 /7  (.10) | 7 / 7  (.33) | 7 / 7  (.43) |
| Leg pain intensity (0-10 Numeric Rating scale), median (*P* value) | 2 / 1  (.37)^#^ | 1 / 2  (.06) | 2 / 2  (.67) | 1 / 2  (.22) | 1 / 2  (.10) | 1 / 2  (.38) |
| Roland-Morris Disability Questionnaire proportional sum score (0-100=highest disability), median (P value) | 57 / 52  (.36)^#^ | 57 / 52  (.67) | 52 / 57  (.68) | 52 / 57  (.62) | 57 / 52  (.63) | 52 / 57  (.66) |
| Recovery belief (0-10=very likely), median [10%; 25% percentile], (*P* value) | 9 [4;7] /  9 [4;6]  (.37)^#^ | 9 [4;7] /  9 [2;5]  **(.02)** | 9 [4;7] /  9 [2;5]  **(.01)** | 9 [4;7] /  9 [2;5]  (.06) | 9 [4;7] /  9 [2;5]  **(.02)** | 9.5 [4;7] /  9 [2;5]  **(.01)** |
| **Latent Class Analysis derived subgroupings** |  |  |  |  |  |  |
| **Single-stage subgroups, *P* value**** | .92^¤^ | .24 | **.01** | .26 | .19 | **.01** |
| SS1 (mild) | 17% / 16% | 17% / 16% | 17% / 15% | 17% / 14% | 17% / 16% | 17% / 16% |
| SS2 (recent disability) | 21% / 20%/ | 22% / 17% | 23% / 15% | 21% / 18% | 22% / 17% | 23% / 14% |
| SS3 (work-related) | 13% / 15% | 13% / 17% | 13% / 18% | 14% / 14% | 13% / 17% | 12% / 18% |
| SS4 (nerve root) | 8% / 8% | 9% / 7% | 9% / 6% | 8% / 8% | 9% / 6% | 9% / 7% |
| SS5 (very recent) | 15% / 14% | 15% / 13% | 15% / 14% | 15% / 12% | 15% / 13% | 15% / 14% |
| SS6 (persistent) | 13% / 16% | 14% / 15% | 13% / 19% | 13% / 19% | 14% / 15% | 13% / 18% |
| SS7 (severe) | 12% / 12% | 11% / 16% | 11% / 13% | 11% / 15% | 11% / 16% | 11% / 13% |
| **Two-stage subgroups, *P* value**** | .72^¤^ | .20 | **.04** | .51 | .20 | **.01** |
| TS1 (mild) | 17% / 18% | 19% / 13% | 19% / 13% | 18% / 15% | 18% / 13% | 19% / 12% |
| TS2 (mild, work issues) | 7% / 10% | 7% / 10% | 8% / 7% | 8% / 8% | 8% / 10% | 8% / 8% |
| TS3 (mild, sleep well) | 8% / 6% | 7% / 8% | 8% / 7% | 8% / 6% | 7% / 8% | 8% / 7% |
| TS4 (persistent) | 5% / 4% | 5% / 4% | 5% / 4% | 5% / 5% | 5% / 4% | 5% / 4% |
| TS5 (sleep issues) | 13% / 11% | 13% / 9% | 13% / 9% | 13% / 8% | 13% / 8% | 14% / 8% |
| TS6 (work-related) | 13% / 15% | 13% / 17% | 12% / 18% | 13% / 15% | 13% / 17% | 12% / 18% |
| TS7 (nerve root) | 5% / 5% | 5% / 6% | 5% / 6% | 5% / 8% | 5% / 6% | 5% / 6% |
| TS8 (very recent) | 24% / 22% | 23% / 26% | 22% / 27% | 23% / 26% | 23% / 26% | 22% / 28% |
| TS9 (severe) | 7% / 8% | 7% / 10% | 7% / 9% | 8% / 8% | 7% / 10% | 7% / 10% |
| Bold indicates statistical significance, *P* < .05  *Missing values were excluded when calculating the p-values  **Association between the prognostic model and follow-up responder status  ^¤^χ^2^ test  ^#^ Wilcoxon rank-sum test | | | | | | |

## Table A4. Test of differences between compliant responders and non-compliant responders* on the SMS questions over 12 months

| **Outcome variables**  **Baseline characteristics** | **Back pain intensity the last week** | **Days with activity limitation the last week** | **Days with LBP the last week** |
| --- | --- | --- | --- |
| Males (*P* value) | 54% / 59%  (.23)^¤^ | 54% / 58%  (0.38) | 54% / 58%  (0.31) |
| Age, median (*P* value) | 43 / 43  (.77)^#^ | 43 / 43  (.94) | 43 / 42  (.62) |
| STarT Back Tool, *P* value** | **.01**^#^ | **.01** | **.01** |
| Low risk group | 56% / 45% | 56% / 45% | 56% / 45% |
| Medium risk group | 36% / 46% | 36% / 46% | 36% / 46% |
| High risk group | 8% / 9% | 8% / 9% | 8% / 9% |
| Quebec diagnostic classification, *P* value | .53^¤^ | .53 | .57 |
| Local LBP | 67% / 64% | 67% / 64% | 67% / 64% |
| LBP + leg pain above the knee | 24% / 25% | 24% / 25% | 24% / 24% |
| LBP + leg pain below the knee | 7% / 10% | 7% / 10% | 7% / 10% |
| LBP + leg pain and neurological signs | 2% / 2% | 2% / 2% | 2% / 2% |
| Back pain intensity (0-10 Numeric Rating Scale), median (*P* value) | 7 / 7  (.13)^#^ | 7 / 7  (.14) | 7 / 7  (.23) |
| Leg pain intensity (0-10 Numeric Rating Scale), median (*P* value) | 1 / 2  (.07)^#^ | 1 / 2  **(.04)** | 1 / 2  (.06) |
| Roland-Morris Disability Questionnaire proportional sum score (0-100=highest disability), median (*P* value) | 52 / 57  (.80)^#^ | 52 / 57  (.69) | 52 / 57  (.83) |
| Recovery belief (0-10=very likely), median [10%;25% percentile], (*P* value) | 9 [4;7] / 9 [2;5]  (.08)^#^ | 9 [4;7] / 9 [2;5]  (.10) | 9 [4;7] / 9 [2;5]  **(.05)** |
| **Latent Class Analysis derived**  **subgroupings** |  |  |  |
| **Single-stage subgroups, *P* value**** | **.01**^¤^ | **.01** | **.01** |
| SS1 (mild) | 17% / 17% | 17% / 17% | 16% / 17% |
| SS2 (recent disability) | 22% / 14% | 22% / 14% | 23% / 13% |
| SS3 (work-related) | 13% / 17% | 13% / 17% | 13% / 17% |
| SS4 (nerve root) | 9% / 5% | 9% / 5% | 9% / 5% |
| SS5 (very recent) | 15% / 13% | 15% / 12% | 15% / 13% |
| SS6 (persistent) | 13% / 19% | 13% / 19% | 13% / 20% |
| SS7 (severe) | 11% / 15% | 11% / 16% | 11% / 15% |
| **Two-stage subgroups, *P* value**** | .33^¤^ | .33 | .23 |
| TS1 (mild) | 18% / 15% | 18% / 15% | 18% / 14% |
| TS2 (mild, work issues) | 8% / 7% | 8% / 7% | 8% / 7% |
| TS3 (mild, sleep well) | 8% / 6% | 8% / 6% | 8% / 6% |
| TS4 (persistent) | 5% / 4% | 5% / 4% | 5% / 4% |
| TS5 (sleep issues) | 13% / 9% | 13% / 9% | 13% / 9% |
| TS6 (work-related) | 13% / 16% | 13% / 16% | 13% / 17% |
| TS7 (nerve root) | 5% / 5% | 5% / 5% | 5% / 5% |
| TS8 (very recent) | 23% / 28% | 23% / 28% | 23% / 27% |
| TS9 (severe) | 7% / 10% | 7% / 10% | 7% / 11% |
| Bold indicates statistical significance, *P* < .05  *Noncompliant SMS responders had 10 or more weeks with missing SMSs  ******Association between the prognostic model and responder status  ^¤^χ^2^ test  ^#^Wilcoxon rank-sum test | | | |
